# Identifying conditions for a third dose intention of COVID-19 vaccination in college students: A fuzzy-set qualitative comparative analysis

**DOI:** 10.3389/fpubh.2022.932243

**Published:** 2022-08-12

**Authors:** Wanqin Gao, Yulin Zhang, Gang Yin

**Affiliations:** ^1^Department of Social Work, School of Literature, Law and Economics, Wuhan University of Science and Technology, Wuhan, China; ^2^Department of Health Administration, School of Medicine and Health Management, Tongji Medical College, Huazhong University of Science and Technology, Wuhan, China

**Keywords:** vaccine hesitancy, vaccination intention, COVID-19 vaccine, college students, qualitative comparative analysis

## Abstract

**Background:**

During the pandemic, a third dose of the COVID-19 vaccine effectively reduces the proportion of severe cases in those infected, but vaccine hesitancy impedes this reasonable prevention method. Epidemic control in China is being tested due to the large population base, especially in crowded places like college campuses. This study aimed to explore the configuration paths of psychological antecedents for college students to receive a third COVID-19 vaccine.

**Methods:**

An anonymous cross-sectional survey was carried out in five universities in Wuhan using convenience sampling. A long version of the 5C 7-point Likert scale was used to measure college students' intention and psychological antecedents on the third dose of the COVID-19 vaccine. A fuzzy-set Qualitative Comparative Analysis (QCA) approach was performed to explore the configuration of conditions to the vaccination willingness.

**Results:**

31.67% of respondents surveyed did not receive their third dose of the COVID-19 vaccine. The score of intention to get the vaccine for college students who did not receive the booster vaccine was 4.93 (±1.68). Average scores of 5.19 (±1.24), 4.35 (±1.75), 4.02 (±1.45), 5.84 (±1.23), and 4.61(±1.32) were reported for confidence, complacency, constraints, calculation, and collective responsibility in them. QCA showed high confidence and collective responsibility playing a central role in third dose vaccination intention. Meanwhile, low confidence and collective responsibility are the core conditions of low vaccination willingness.

**Conclusion:**

Eliminating vaccine hesitancy necessitates focusing on the psychological antecedents of vaccination intentions to identify critical targets for policy and interventions. This study identified trust and collective responsibility are core elements of the psychological antecedents of college students' intention to receive the booster vaccine for COVID-19. To achieve herd immunity as soon as possible, health administration and campus can start with vaccine confidence-building and collective responsibility cultivation to take appropriate actions and measures to improve coverage of the booster vaccination.

## Introduction

The COVID-19 pandemic has caused worldwide social, economic, and educational devastation, severely affecting people's health and livelihoods. Evidence-based medicine suggests that COVID-19 vaccination is the most logical intervention for pandemic control (1). However, the effectiveness of the COVID-19 vaccine has declined over time, along with the continued spread of the SARS-CoV-2 virus and the emergence of its variants (2).

Therefore, WHO stated in March 2022 strongly supporting the urgent and extensive vaccination of booster doses to address the highly infectious Omicron strain (3). In China, the current situation of prevention remains dire for a country of 1.4 billion citizens after multiple cases of imported SARS-CoV-2 variant strains from abroad have triggered local outbreaks since 2021. 2022, China's National Health Commission proposed in the Treatment Protocol for Novel Coronavirus Infection (9th edition) that vaccine recipients eligible for vaccination should receive a booster dose promptly and encouraged residents to receive booster vaccinations to improve protection against SARS-CoV-2 virus and promote herd immunity (4). Vaccination is the safest and fastest method to achieve herd immunization, and reaching this target requires at least 70–90% of the population to be vaccinated (5, 6). Independent Allocation of Vaccines Group (IAVG)'s call for 70% COVID-19 vaccine coverage in all countries by 2022 is imperative, but vaccine hesitancy remains a global challenge (7).

Vaccine hesitancy is the delay or refusal of vaccination despite the availability of vaccination services. It is complex and varies with time, place, and vaccine type. In 2019, WHO listed vaccine hesitancy as one of the 10 threats to global health. Several studies have shown that COVID-19 vaccine hesitancy is common in college student populations, as demonstrated in studies in the United States, Switzerland, Japan, Arab Emirates, Italy, Czech Republic, France, and other countries (8–15). University campuses are densely populated and bring together people from different places. Also, college students are socially active and in close contact, and there is a high risk of infection for coronavirus. In addition, college students are generally highly independent. They have a unique judgment about the inoculation perception, may be stubborn, and fall into the misunderstanding of hesitancy or rejection of the vaccine. Therefore, to achieve herd immunity as soon as possible, it is essential to eliminate hesitancy among the student population regarding the third dose of the COVID-19 vaccine.

Because vaccine hesitancy describes a psychological state in the middle between complete acceptance and complete rejection of vaccines, there are many theoretical models in psychology used to predict the generation of vaccine hesitancy. Strategic Advisory Group Experts (SAGE) proposed the 3C model of vaccine hesitancy, identifying three key factors of vaccine hesitancy: complacency (not believing that the disease is high-risk and vaccination is necessary), convenience (practical barriers), and confidence (lack of trust in the safety and efficacy of the vaccine) (16). Betsch developed the 4C model, which adds to the 3C by adding calculation (individual involvement in extensive information search) as an additional psychological antecedent to the 3C model (17). After that, based on the Health Belief Model and the Theory of Planned Behavior (18, 19), the 5C model of psychological antecedents for vaccination intention was developed by Betsch in 2019. It also builds on established theoretical vaccine hesitancy and acceptance models and relates them to psychological models explaining health behaviors (16, 17, 19–21).

Furthermore, compared Vaccine Confidence Scale (VCS), Global Vaccine Confidence Index (GVCI), Vaccine Hesitancy Scale (VHS), Vaccine Confidence Index (VCI), Vaccine Acceptance Scale (VAS), and other scales measuring vaccine hesitancy, the 5C scale demonstrated high reliability and validity in cross-cultural regions (22). As a new measure used to capture relevant predictors of vaccination behavior, the 5C scale's greatest strength lies in its relationship to theory and its empirical association with psychological constructs. The 5C scale can measure the psychological antecedents of vaccination behavior in college students with COVID-19 vaccination and assess the relative importance of psychological antecedents for further exploration (22).

There is still a gap in research on the willingness and factors influencing college students to receive a third dose of the COVID-19 vaccine, especially in a country with a large population like China. Many meaningful explorations have been made by scholars on the topic of COVID-19 vaccine hesitancy (23–29). However, most of them have explored the factors leading to vaccine hesitancy using statistical methods such as regression models, which are symmetrical research methods focusing on the “net effect” of variables and have not yet revealed the complex mechanism of multi-factor interaction (10, 11, 14). However, the degree of vaccination intention is a multifactorial outcome with multiple causal pathways, and the combined effect of antecedent and causal variables should be considered.

Based on the 5C (Confidence, Complacency, Constraints, Calculation, and Collective responsibility) model of psychological antecedents of vaccination developed by Betsch (22), this study explored the configuration of the third dose of the vaccine among college students in Wuhan by using fuzzy-set Qualitative Comparative Analysis (QCA). Our study sheds new light on college students' willingness and psychological antecedents to receive a booster dose of the COVID-19 vaccine in the city, with the largest number of college students globally. This study can help policymakers better understand the psychological antecedents of booster vaccination for university students, thereby facilitating government efforts to convert willingness to receive a booster shot into campaigns to counteract the epidemic through health education and policy advocacy.

The rest of the study is organized as follows. Section 2 introduces the theoretical basis and analytical methods of this study and describes the sample, indicators, and data sources. Section 3 reports the results of the measurement of college students' willingness to receive the third dose of COVID-19 vaccine in Wuhan and the critical steps of the fuzzy-set QCA, including calibration, necessity analysis, configuration analysis, and robustness test. Section 4 summarizes these findings and clarifies the highlights of this study.

## Methods and materials

### Study design and data collection

The questionnaire consisted of two parts, the first part was demographic information, and the second part was a 5C scale of psychological antecedents of vaccination intention. This anonymous cross-sectional survey was conducted in Wuhan, China, using a convenience sampling method from March to April 2022. Five sampling sites included different categories of universities (comprehensive university, liberal arts university, polytechnic university, medical college, and vocational & technical college). The target group of this research is college students. To maintain social distance during the pandemic, respondents filled out the questionnaire online by scanning the QR code in the survey poster and filling out the electronic questionnaire on the Wenjuanxin platform (Changsha Ranxing Information Technology Co., Ltd., Changsha). To ensure the integrity of data acquisition, the questionnaire could not be submitted if there were missing values. All participants confirmed the electronic informed consent form before the questionnaire was completed and could opt out of responding at any time after the questionnaire was started. If the respondents do not understand the questionnaire's contents, the survey members will explain it. The members received uniform training to ensure consistency of answers when interpreting the questionnaire. No expenses were paid to the participants in this survey. The study was conducted according to the guidelines of the Declaration of Helsinki and was approved by the Department of Social Work, School of Literature, Law and Economics, Wuhan University of Science and Technology.

This questionnaire consists of 25 items. A pre-survey of 30 samples was conducted before the official survey. The questionnaire was revised and improved based on the pre-survey results for unclear presentation, and misleading and redundant text problems. Many campuses were locked down due to the Omicron outbreak. A total of 502 questionnaires were distributed, all of which were valid.

### Measures

#### Intention for a third dose vaccination

Intention to receive the third dose of COVID-19 vaccine among university students was measured on a 7-point Likert scale, and the question was set as follows: *what is your willingness to receive the third dose of COVID-19 vaccine?* This was the tenth question in the questionnaire, and the level of desire to receive the vaccine ranged from “very reluctant” to “very willing”.

#### Psychological antecedents

A validated 5C scale was used to measure the psychological antecedents of the third dose of the COVID-19 vaccine among college students. These antecedents interact with each other and then affect the willingness to vaccination to varying degrees. The 5C scale is named after the initial letter C of each antecedent condition. They include confidence, complacency, constraints, calculation, and collective responsibility (30). As shown in Table 1, the long version of the scale consists of 15 questions, each C consisting of 3 sub-items, with answers in the form of a Likert scale of 7 levels ranging from strongly disagree to agree strongly.

**Table 1:** Items of 5C scale measuring psychological antecedents of the third dose of COVID-19 vaccine.

**5C**	**Number**	**Items**
Confidence	1	I am entirely confident that the third dose of the COVID-19 vaccine is safe.
	2	The third dose of COVID-19 vaccination is effective.
	3	Regarding the third dose vaccination, I am confident that public authorities decide in the best interest of the community.
Complacency	4	The third dose of vaccination is unnecessary because COVID-19 is not common anymore.
	5	My immune system is so strong that it also protects me against COVID-19.
	6	COVID-19 is not so severe that I should get a third dose vaccination.
Constraints	7	Everyday stress prevents me from getting a third dose COVID-19 vaccination.
	8	For me, receiving the third dose of COVID-19 vaccinations is inconvenient.
	9	Visiting the doctor makes me uncomfortable, this keeps me from getting a third dose of COVID-19 vaccination.
Calculation	10	When I consider getting a third dose COVID-19 vaccination, I weigh the benefits and risks to make the best decision possible.
	11	For every vaccination, I closely consider whether it is helpful for me.
	12	It is essential for me to fully understand the topic of a third dose COVID-19 vaccination before I get vaccinated.
Collective Responsibility	13	I don't have to get vaccinated when everyone gets a third COVID-19 vaccine.
	14	I get a third dose of the COVID-19 vaccine because I can also protect people with a weaker immune system.
	15	The third dose of vaccination is a collective action to prevent the spread of diseases.

As with the English version of the scale, the questionnaire required reverse coding for the score of question 13, which reads: *I don't have to get vaccinated when everyone gets a third COVID-19 vaccine*. In this study, reliability analysis was performed using Cronbach's α. The Cronbach's α of each dimension in the 5C scale was 0.917, 0.873, 0.702, 0.865, and 0.671, indicating good reliability. The scale's content validity was evaluated by six experts (majoring in psychology, sociology, and healthcare) in this study. For the Item-level Content Validity Index (I-CVI), the scale was scored on a 4-point scale, with the I-CVI taking values from 0.83 to 1.00. And Scale-level Content Validity Index (S-CVI) Average was 0.915.

### Qualitative comparative analysis approach

The psychological antecedents of college students' third dose of the COVID-19 vaccine combine and thus influence vaccination intentions. Most extant studies use regression models to explore the net effect of a single factor on vaccination outcomes, making it difficult to explain the multiple causal relationships between antecedent conditions and vaccination willingness. While traditional regression models emphasize symmetry between conditions and consequences when exploring factors influencing vaccine hesitancy. But the reality is that leading to high vaccination intention is often inconsistent with factors that produce low vaccination intention and are asymmetric.

The willingness of college students to receive the third dose of the COVID-19 vaccine is an outcome. Still, the antecedent conditions and their combinations that lead to high or low willingness are complex and diverse, which is a typical multiple concurrent causation problem. As a new method beyond qualitative and quantitative, QCA can explore this complex causal relationship. QCA is based on set-theoretic ideas and Boolean operations to examine the conditional histories that lead to the outcome from a holistic and systematic perspective, with three main types, clear-set (cs-QCA), multi-valued set (mv-QCA), and fuzzy-set (fs-QCA) (31, 32). Because college students' willingness to receive a third dose of the COVID-19 vaccine is between strong willingness and reluctance, i.e., vaccine hesitation, which meets the application conditions of fuzzy-set QCA. From the set theory perspective, the pathways leading to high and low intention vary, and different antecedent conditions can lead to different outcomes. For example, some groupings have high coverage and form the primary grouping pathway for vaccination intention, while others have low coverage and become secondary pathways for vaccination intention. However, regardless of the difference in coverage, all configuration paths led to the same outcome, the same as the meaning of all roads leading to Rome.

Therefore, in this study, the fuzzy-set QCA (fs-QCA) method was used to analyze college students' psychological antecedent histories to receive the third dose of the COVID-19 vaccine. To our knowledge, few studies have applied this approach to discuss the factors influencing vaccine hesitancy. In addition, the QCA method is particularly suitable for case investigation of small and medium-sized samples, which is ideal for the population of this study. These, i.e., university students, have not yet received their third vaccination. After all, in the context of universal vaccination with the COVID-19 vaccine, the group that has not yet been vaccinated is still a minority.

## Results

### Participants characteristics

A total of 502 respondents participated in this survey, including 321 females (63.9%) and 181 males (36.1%). 139 (27.7%) of them were first-year students, 212 (42.2%) were sophomores, 121 (24.1%) were juniors, and 30 (6%) were seniors. A total of 283 undergraduates (56.4%) of the total number of participants, college students were 83 (16.5%), and graduate students and above were 136 (27.1%); 199 (39.6%) of the students served as student cadres, and 9.4% had a history of chronic diseases. Of the survey results, 340 (67.7%) had received their third dose of COVID-19 vaccine, 159 (31.7%) participants reported having received only two doses of COVID-19 vaccine but no third dose, and three participants may not have received any amount of COVID-19 vaccine; the demographic characteristics of all respondents are shown in Table 2.

**Table 2:** Participant characteristics.

**Variables**	**Target group (159)**		**Total (502)**	
**Gender**	**n**	**%**	**n**	**%**
Male	60	37.70	181	36.10
Female	99	62.30	321	63.90
**Educational level**				
Junior college student	27	17.00	83	16.50
Undergraduate student	77	48.40	283	56.40
Graduate student and above	55	34.60	136	27.10
**Grade**				
First-year	41	25.80	139	27.70
Second-year	58	36.50	212	42.20
Third-year	43	27.00	121	24.10
Fourth-year and above	17	10.70	30	6.00
Student cadres	62	39.00	199	39.60
Medical-related majors	35	22.01	58	11.55
Chronic case history	21	13.20	47	9.40
Chronic medical history of family members	79	49.69	205	40.84
**5C vaccination antecedents**	**Mean**	**SD**	**Mean**	**SD**
Confidence	5.19	1.24	5.64	1.12
Complacency	4.35	1.75	3.27	1.75
Constraints	4.02	1.45	4.26	1.96
Calculation	5.84	1.23	5.95	1.20
Collective responsibility	4.61	1.32	5.45	1.31
**The third dose vaccination intention**	***n***	**%**	***n***	**%**
1	0	0.00	1	0.20
2	12	7.50	17	3.40
3	27	17.00	31	6.20
4	29	18.20	45	9.00
5	31	19.50	63	12.50
6	12	7.50	117	23.30
7	48	30.20	228	45.40
Mean	4.93	-	5.82	-
SD	1.68	-	1.43	-

Target group means the college students who have received the first and second dose of the COVID-19 vaccine but have not received the third dose. Vaccination intention was divided into seven levels, 1 means very reluctant and 7 means very willing. n represents the number of observations. SD represents standard deviation.

Of these, 159 students who had received the first two doses of the COVID-19 vaccine but had not yet received a booster dose were the focus of this study, either because they had not yet met the conditions for a COVID-19 vaccine booster (e.g., <6 months since the second dose) or because of vaccine hesitancy. However, in any case, understanding their willingness to vaccinate and the psychological antecedents of vaccination is necessary to achieve the goal of herd immunity. There were 99 (62.3%) females, and 60 (37.7%) males among those who did not receive the booster shot, of whom 41 (25.8%) were first-year students, 58 (36.5%) were sophomores, 43 (27%) were juniors, and 17 (10.7%) were seniors. The descriptive statistical analysis of vaccination intention and psychological antecedent scores of 159 college students is shown in Table 3.

**Table 3:** Descriptive statistics of antecedent and outcome variables.

**Variables**	**Mean**	**Median**	**SD**	**Max**	**Min**
Intention	4.93	5	1.68	7	2
Confidence	5.19	5	1.24	7	2
Complacency	4.35	4.67	1.75	7	1
Constraints	4.02	4	1.45	7	1
Calculation	5.84	6.33	1.23	7	2
Collective responsibility	4.61	4.67	1.32	7	2

### Variables calibration

Fuzzy sets require converting all metric scores into sets, and qualitative anchor points determine the relationship between continuous variable scores and fuzzy set affiliations (33). The calibration function in the fs-QCA software is applied to calibrate each variable. The variables were converted into calibration sets by setting thresholds; full membership, full non-membership, and crossover points (33). The calibration set describes whether the case is more in or out of the set (31). Psychological antecedent variables and vaccination intention scores for college students to receive the COVID-19 vaccine booster were calibrated using a 7-point Likert scale, following Ordanini's approach (34). The fully affiliated anchor points were set to 6, the crossover points to 4, and the fully unaffiliated anchor points to 2. At the end of the calibration, the calibration results were modified concerning Greckhamer's study to avoid the crossover values being ignored in the calculation and thus affecting the analysis results (35). This was done by increasing the affiliation value by 0.001 for the presence of 0.5 and modifying it to 0.501.

### Necessity analysis

Necessity analysis explores the extent to which the set of outcomes constitutes a subset of the set of conditions. If a condition always appears when some outcome is present, then this condition is necessary for the existence of the outcome. Before conducting the standard analysis, the relationship between each condition variable and the outcome variable needs to be examined to analyze whether each condition variable is necessary for the outcome variable. Necessary condition analysis was performed by fs-QCA software to test the consistency and coverage of each antecedent variable. The consistency value is an essential indicator of the necessary conditions. The lowest value of the consistency score for the condition of necessity is usually considered to be 0.9 (33). As shown in Table 4, when the outcome variable was high vaccination intention, the consistency of the antecedent variable of confidence was 0.9650, while the consistency of all other antecedent variables was <0.9, implying that high confidence may be a necessary condition for the high intention of college students to receive the booster shot of the COVID-19 vaccine. In addition, in the test of necessity for low vaccination intention, the consistency of both variables, complacency, and calculation, was > 0.9, implying that calculation and complacency may be necessary for college students' hesitancy to receive a third dose of the COVID-19 vaccine.

**Table 4:** Analysis of necessary conditions.

**Antecedent variables**	**High intention**		**Poor intention**	
	**Consistency**	**Coverage**	**Consistency**	**Coverage**
Confidence	0.9650	0.8428	0.6980	0.3194
~ Confidence	0.2207	0.5824	0.6564	0.9076
Complacency	0.5882	0.6337	0.9110	0.5144
~ Complacency	0.5492	0.9218	0.3512	0.3089
Constraints	0.5783	0.7628	0.7095	0.4905
~ Constraints	0.6137	0.8013	0.6569	0.4494
Calculation	0.8679	0.6686	0.9909	0.4000
~ Calculation	0.2212	0.9789	0.1790	0.4152
Collective responsibility	0.8489	0.8904	0.5687	0.3126
~ Collective responsibility	0.3447	0.6040	0.8006	0.7352

**Figure 1:**
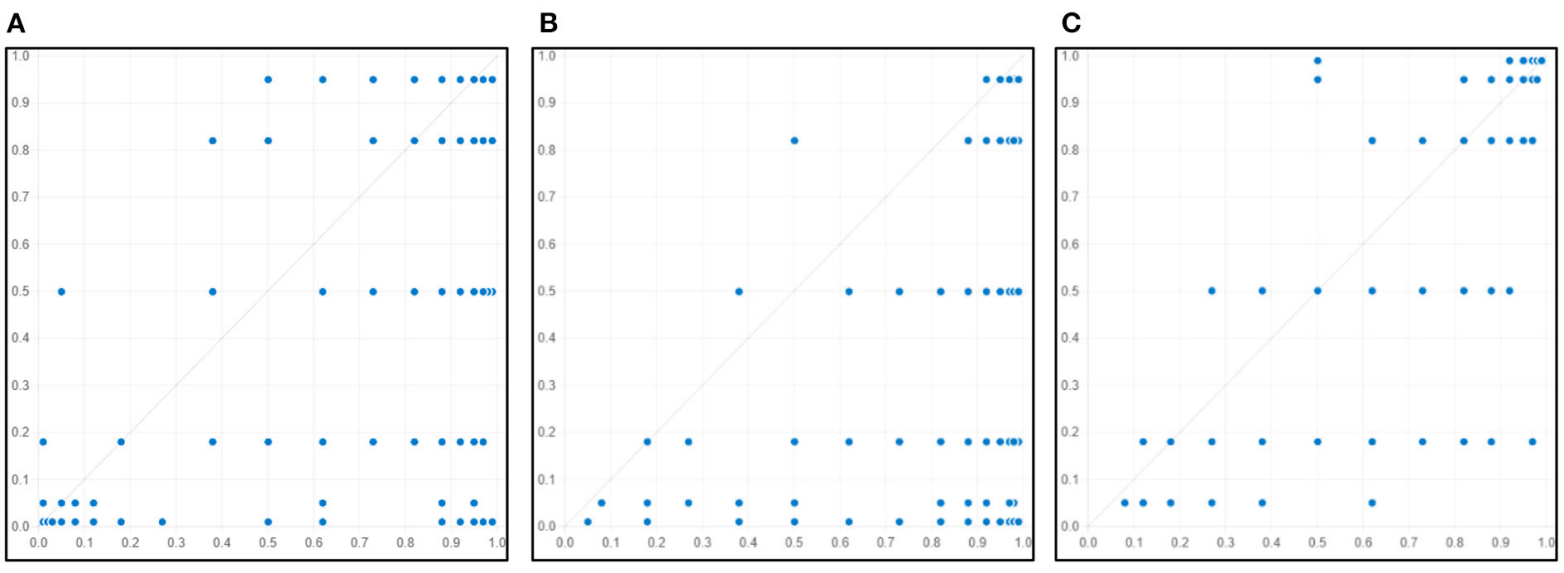
Distribution of cases for necessary condition X (X-Y plot). **(A)**: X means complacency, Y means poor intention. **(B)**: X means calculation, Y means poor intention. **(C)**: X means confidence, Y means high intention.

Following Schneider's study, when a condition constitutes the necessary condition for the occurrence of the result, it needs to show consistency > 0.9 and have non-trivial coverage (33). When the result is low willingness, the coverage of complacency and calculation is low, so it can be judged that they are not necessary conditions for low willingness. In addition, by plotting the X-Y scatter plots between the three possible necessity conditions and the outcome variables, cases were found to be present above the diagonal. Specifically, Figure 1A shows that many cases are distributed on the right side of the vertical axis, so high complacency is also not necessary for low willingness. Figure 1B has cases distributed on the right side of the vertical axis, meaning that high calculation is unnecessary for low willingness to vaccinate. Figure 1C finds that many cases are distributed above the diagonal, indicating that high confidence is not necessary for high intention to vaccinate.

### Configuration analysis

Based on the calibration and necessity analysis results, a standard analysis of the conditions under which the results were generated was performed. A data matrix (truth table) containing 2^5^ rows was constructed. Each row of the matrix was associated with a specific combination of attributes and a large number of cases (31). We set the frequency of the minimum solution to be set to 2 and the consistency threshold of the solution to be chosen to be 0.8, depending on the number of included samples (33). In addition, to avoid groups with PRI thresholds below 0.5 exhibiting significant inconsistencies (35). Therefore, the PRI consistency threshold for this study was set to 0.7. Three solutions, namely complex, parsimonious and intermediate solutions, were obtained for high and low vaccination intention for the third dose of the COVID-19 vaccine after the standard analysis.

As shown in Table 5, two histories (C1 and C2) can explain the high willingness of college students to receive the booster shot, while C3 can explain the low desire of college students to receive the booster shot. A black circle indicates the presence of the condition. A crossed circle indicates spaces indicating the lack of the condition. The blank cells represent conditions that do not matter for the solution. Large circles indicate core conditions where the outcome exists and small circles indicate marginal or auxiliary conditions where the outcome exists (36). The relevant parameters for each configuration path, such as original coverage, unique coverage, and overall solution coverage, are described in Table 5. According to Ragin and Fiss's studies, the original coverage is the proportion of cases that satisfy this configuration. The unique coverage is the proportion of cases that uniquely satisfy this configuration but not any other configuration (31, 36).

**Table 5:** Configuration of conditions for the third dose of COVID-19 vaccination intention.

**Configuration**	**High intention**		**Low intention**
	**C1**	**C2**	**C3**
Confidence	⚫	⚫	⭙
Complacency			•
Constraints		⊗	
Calculation	•		•
Collective responsibility	⚫	⚫	⊗
Consistency	0.9003	0.9248	0.9383
Raw coverage	0.7538	0.5518	0.6122
Unique coverage	0.2713	0.0693	0.6122
Overall solution consistency	0.8231		0.9383
Overall solution coverage	0.9068		0.6122

Full black circles and crossed-out circles indicate the presence and the absence of causal conditions, respectively. Large circles (⚫ and ⊗) indicate the core conditions, small circles (• and ⊗) indicate the peripheral conditions, and the blank cells represent conditions that do not matter for the solution.

### Robustness test

The robustness test is an essential step in QCA, and this study followed Schneider's method to test the robustness of QCA results by changing the consistency level (33). When the consistency level is changed from 0.8 to 0.85, the group results are the same as in Table 5. Also, we adjusted the case frequency from 2 to 3 based on the consistency being 0.85 and selected a more stringent restriction threshold. The results of the criterion analysis show that the adjustment makes the parsimonious solution more streamlined, changing from high confidence and high collective responsibility to high collective responsibility. In contrast, the intermediate solution has only a slight change. Compared with the initial grouping, key parameters such as the number of groups, solution consistency, and coverage did not show substantial changes, so the QCA analysis results were considered relatively reliable.

## Discussion

To the best of our knowledge, this is the first study to explore college students' willingness to receive the third dose of the COVID-9 vaccine and its psychological antecedents by using fs-QCA. We found that two combinations of conditions (i.e., configurations) existed to achieve high intention to receive a third dose of the COVID-19 vaccine among college students. Two configurations exhibited second-order equivalence, i.e., the core conditions were identical.

The consistency of the solution in C1 (Confidence ^*^ Collective Responsibility ^*^ Calculation) was 0.9003, exhibiting acceptable consistency (≥ 0.8). These conditions suggest that among the psychological antecedents of college students' willingness to vaccinate with the third dose of the COVID-19 vaccine, the presence of three psychological factors, high confidence, high collective responsibility, and high calculation, can lead to a high willingness to vaccinate, regardless of the presence of constraints and complacency. Increased confidence and high collective responsibilities are the core conditions, and high calculation is the peripheral condition. The core conditions are those that have a significant impact on the results, and the peripheral conditions are those that make a secondary contribution. This pathway is consistent with the critical factors derived by Dratva et al. using logistic regression models, namely the importance of confidence and collective responsibility in COVID-19 vaccination intention (10). Confidence describes the level of trust individuals have in the efficacy and safety of the vaccine and the health care system and government decisions. In this survey, the mean score of 159 respondents on the confidence dimension was 5.19, with scores of 5.83, 4.88, and 6.21 in the three sub items of this dimension. The third sub item contributed the highest score in this dimension. This sub-item reads that the decision to allow everyone to receive the third dose of the COVID-19 vaccine was made by the authorities in the best interest of the people. The score of this item shows the high level of trust that college students have in the government. In 2020, China was ranked first on the global trust index of the Edelman Trust Barometer, one of the few countries with a large population that maintained high confidence in its government during the pandemic (37).

At the same time, collective responsibility describes the willingness to protect others through one's vaccination, reflecting a sense of collective responsibility. Under the dimension of collective responsibility, the mean scores of the three sub-items were 3.28, 5.6, and 6.04, respectively. Item, I think it is my collective responsibility to prevent the spread of the Coronavirus by receiving the third dose of the COVID-19 vaccine, scoring the highest, probably because the college student group has a higher level of education and better understands the importance of mass immunization. In addition, calculation indicates the extent to which individuals actively search for information, a dimension that reflects the motivation to think about and question vaccination, and those with high calculated scores tend to be less willing to vaccinate (17). Very interestingly, it seems to see a different result in our results, where high calculation turns out to be one of the factors of the psychological antecedent combination of high willingness to vaccinate, given the two core conditions of increased confidence and high collective responsibility, probably because students are more data literate than the general population, and public health institutions and media are their main channels to search for information related to COVID-19 (27). In other words, the information about epidemic prevention and control in the CDC and responsible news media is trustworthy. Encouraging people to be vaccinated still dominate the public media. Not only that, but this particular finding also suggests that analyzing the effects of single factors on intention alone may be inadequate and that analysis of groups of psychological antecedents better explains complex causal relationships. Policymakers can focus on improving confidence and collective responsibility rather than finding ways to reduce calculation aspects.

The consistency of the solution in C2 (Confidence ^*^ Collective Responsibility ^*^ ~ Constraints) is 0.9248. This pathway indicates that the presence of three factors, high confidence, high collective responsibility, and low constraint, can bring about a high willingness to vaccinate college students for the booster dose of the COVID-19 vaccine regardless of the presence complacency and calculation. Confidence and collective responsibility are the core conditions discussed in C1, so in this section, we focus on the marginal condition of low constraint. Among the 159 respondents, the mean scores of the three sub-items under the dimension of constraint were 4.55, 4.52, and 3.7, respectively. The Item, visiting the doctor makes me uncomfortable, this keeps me from getting a third dose of COVID-19 vaccination, scored the lowest and contributed the most to this dimension. This may be related to the vaccination-friendly policies that China has implemented to promote vaccination for the COVID. For example, people can make appointments for COVID-19 vaccinations online, while community health centers set up temporary vaccination sites or provide mobile vaccination vans. Universal PCR testing and vaccination service provision are common in China. Thus it has become the norm for college students to meet medical staff, and people generally feel less pressure. We hypothesize that this sub item will not score high during a pandemic because the diseases that the coronavirus vaccine protects against are different from other vaccines, and the epidemic is a direct threat to the health and safety of the population. From this point of view, the fear of seeing medical staff is less important than obtaining immunity against coronavirus.

Besides, we found only one configuration, C3 (~Confidence, ~Collective Responsibility, Complacency, Calculation), that resulted in low intention to receive a third vaccination dose among college students. This pathway indicates that low confidence, high complacency, high calculation, and low collective responsibility lead to low willingness to receive a booster dose of the COVID-19 vaccine, regardless of constraints. Vaccine hesitancy is the delay or refusal of vaccination despite the availability of vaccination services. It is complex and varies with time, place, and vaccine type (16, 38). The low confidence and low collective responsibility in this configuration are the core conditions of low intention. Low confidence may be due to the influence of false knowledge, where opponents of vaccination present facts and draw wrong conclusions in a one-sided or distorted manner, expecting phenomena that are impossible in science (39). For example, vaccines are required to be free of any side effects. From the perspective of evidence-based medicine, there is no absolutely safe vaccine, and all vaccinations carry some risk (40). Low confidence may also be related to one's health status (41). Some of the vaccine hesitancy in our survey results also have chronic diseases and may be concerned that receiving multiple doses of COVID-19 vaccine will have harmful effects on their already ill bodies, which also suggests that schools and communities should pay more attention to patients with chronic diseases and promptly publicize or popularize which chronic diseases will be affected by vaccination boosters against COVID-19 vaccine. Timely help them determine their suitability for a third dose of vaccine to reduce vaccine hesitation. Low Collective responsibility implies a lack of understanding or awareness of the importance of herd immunization, or it may be a lack of concern or desire to vaccinate others. There is a “free-rider” mentality regarding vaccination or not. Universities have a suitable environment for interaction and can take advantage of campaigns to promote the importance of the third dose of vaccine against coronavirus and the importance of achieving herd immunity. In addition, when boosting herd immunity, students should be motivated to empathize by emphasizing that those who cannot be vaccinated for practical reasons can also be protected. Regular reminders from community health centers and campuses can also play an important complementary role.

In addition, high complacency and high calculation are auxiliary conditions for low intention to receive the third dose of the COVID-19 vaccine. High complacency describes the individual's perceived low risk of disease. Under the dimension of complacency, the three subscales scored 3.15, 3.23, and 3.42, with the highest score for not seriously ill at this stage of infection. Under the national and regional epidemic prevention policy of Prevention of External Importation and Internal Spread (PEIIS), the respondents' locations may be currently free of epidemics or have few epidemics, such as in Wuhan. The fact that omicron only causes mild illnesses, but few severe illnesses and deaths may lead people to take a chance and subjectively believe that the epidemic is not severe. In addition, the variant strain of novel coronavirus causes only mild disease with few illnesses and deaths. However, variant strains, such as Omicron, still put additional pressure on the health care system and may cause considerable disruption to society (42). Therefore, we suggest that linking infection with coronavirus to other diseases for which higher risk perceptions already exist may be a feasible way to raise risk awareness (43). This would allow people to understand and perceive the possible dangerous consequences of removing the epidemic prevention policy. Positive publicity about the loss of health to individuals and the harm to society from an epidemic might increase the population's perception of the high risk of the COVID-19 epidemic.

The high calculation indicates that college students prefer to search for a wide range of information. We speculate that it may be related to the fact that a few students did not seek scientifically helpful information through the proper channels or were influenced by conspiracy theory-related beliefs. As found in Romer's study, social media's information source was associated with COVID-19 conspiracy beliefs and higher vaccine hesitancy (44). Therefore, we call for social media to guide people's perceptions of the COVID-19 epidemic rationally and correctly and suggest that government, community health centers, and schools should regularly promote knowledge about the vaccine booster in social media to provide more scientific channels for students to understand the function of booster vaccination properly. Of course, making full use of the campus environment to hold expert lectures on the third vaccine with proper safety precautions is also considered a feasible way to translate the jargon of booster vaccination into easy-to-understand language for students, thus improving their confidence in the booster.

Accelerated deployment of COVID-19 vaccines and boosters worldwide remains necessary to curb coronavirus spread. The QCA results indicate that low confidence and low collective responsibility are the core conditions of low intention, which is the opposite of the core conditions of high confidence and high collective responsibility for high intention outcomes, showing symmetry. This suggests that the two psychological antecedent variables of confidence and collective responsibility are the most important for the outcome, regardless of whether the willingness is high or low intention. This can provide a basis for policymakers that increasing college students' confidence and collective responsibility can effectively improve the willingness to vaccinate for the third dose of the COVID-19 vaccine, which in turn translates into vaccination behavior.

## Conclusion

Eliminating vaccine hesitancy necessitates focusing on the psychological antecedents of vaccination intentions to identify critical targets for policy and measure interventions. This study explored the psychological antecedents of college students' booster doses of the COVID-19 vaccine in Wuhan city using the QCA approach. Our findings suggest that trust and collective responsibility are core to college students' willingness to receive a third dose of the vaccine, whether high or low. This interesting finding illustrates the importance of boosting college students' confidence in the vaccination coverage and fostering a sense of collective responsibility. To achieve herd immunity as soon as possible, health administration and campuses can start with confidence building in a booster shot and cultivation of collective responsibility to take measures to realize joint immunization as soon as possible to hinder the spread of SARS-CoV-2 virus.

### Strengths and limitations

Previous studies on vaccine hesitancy have used regression methods to explore the key factors that generate vaccine hesitancy. Still, our study focused on the diversity and complexity of the psychological antecedents of vaccination intention, i.e., the combination of different psychological antecedents that lead to high or low intention to receive the third dose of the COVID-19 vaccine among college students. However, this study also has some limitations, such as being affected by the epidemic, failing to use a random sampling method to conduct a cross-sectional survey, and the lack of discussion of the differences in psychological antecedents of willingness to vaccinate between medical students and students of other majors. At the same time, due to different national conditions and cultural environments, it should be cautious about extending the conclusions of this study to other countries outside China. Future studies could start by optimizing the sampling method and comparing the differences in psychological antecedents of willingness to vaccinate across populations. Also, comparing the psychological antecedents of vaccination among college students in different countries will be interesting.

## Data availability statement

The original contributions presented in the study are included in the article/supplementary material, further inquiries can be directed to the corresponding author.

## Ethics statement

The studies involving human participants were reviewed and approved by Department of Social Work, School of Literature, Law and Economics, Wuhan University of Science and Technology. The patients/participants provided their written informed consent to participate in this study.

## Author contributions

WG, YZ, and GY conceived and designed the study. YZ and GY were involved in data collection and analyses. YZ wrote the draft. WG and GY revised the manuscript critically for intellectual content. All authors have read and agreed to the published version of the manuscript. All authors contributed to the article and approved the submitted version.

## Conflict of interest

The authors declare that the research was conducted in the absence of any commercial or financial relationships that could be construed as a potential conflict of interest.

## Publisher's note

All claims expressed in this article are solely those of the authors and do not necessarily represent those of their affiliated organizations, or those of the publisher, the editors and the reviewers. Any product that may be evaluated in this article, or claim that may be made by its manufacturer, is not guaranteed or endorsed by the publisher.
